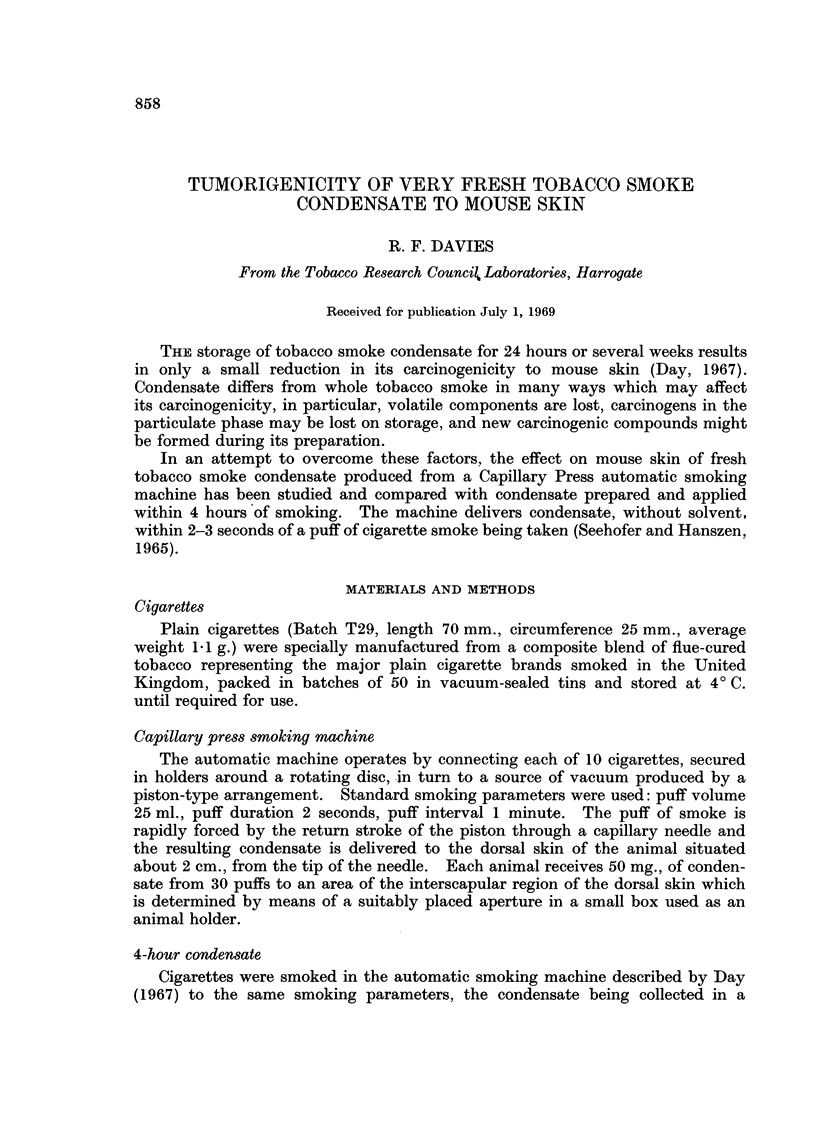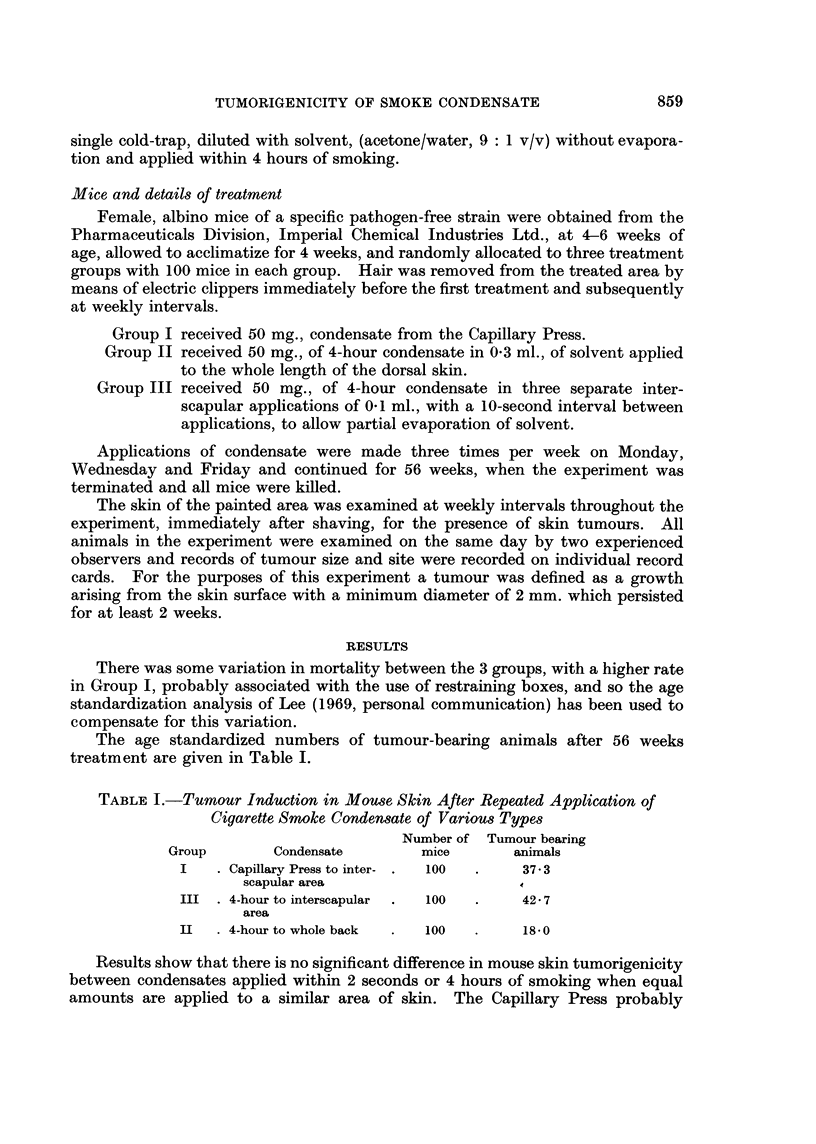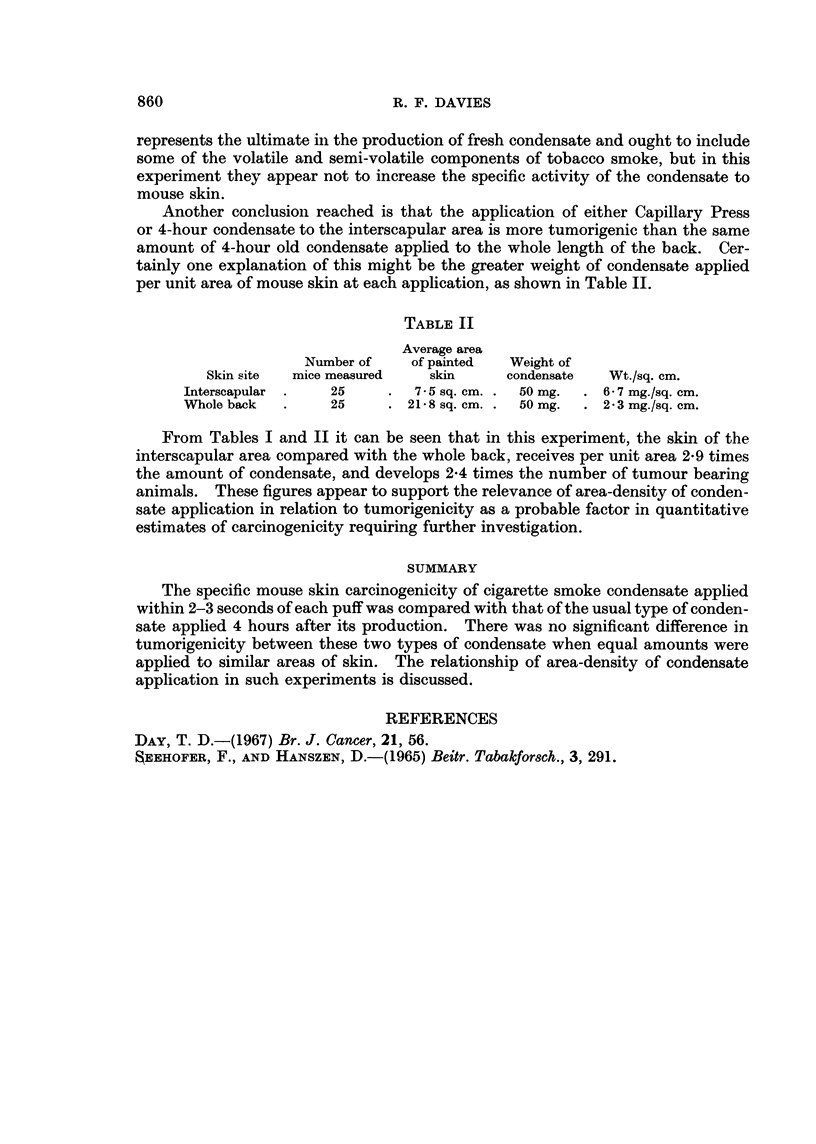# Tumorigenicity of very fresh tobacco smoke condensate to mouse skin.

**DOI:** 10.1038/bjc.1969.105

**Published:** 1969-12

**Authors:** R. F. Davies


### TUMORIGENICITY OF VERY FRESH TOBACCO SMOKE CONDENSATE TO MOUSE SKIN

R. F. DAVIES

*From the Tobacco Research Council Laboratories, Harrogate*

Received for publication July 1, 1969

THE storage of tobacco smoke condensate for 24 hours or several weeks results in only a small reduction in its carcinogenicity to mouse skin (Day, 1967). Condensate differs from whole tobacco smoke in many ways which may affect its carcinogenicity, in particular, volatile components are lost, carcinogens in the particulate phase may be lost on storage, and new carcinogenic compounds might be formed during its preparation.

In an attempt to overcome these factors, the effect on mouse skin of fresh tobacco smoke condensate produced from a Capillary Press automatic smoking machine has been studied and compared with condensate prepared and applied within 4 hours of smoking. The machine delivers condensate, without solvent, within 2–3 seconds of a puff of cigarette smoke being taken (Seehofer and Hanszen, 1965).

### MATERIALS AND METHODS

*Cigarettes*

Plain cigarettes (Batch T29, length 70 mm., circumference 25 mm., average weight 1·1 g.) were specially manufactured from a composite blend of flue-cured tobacco representing the major plain cigarette brands smoked in the United Kingdom, packed in batches of 50 in vacuum-sealed tins and stored at 4° C. until required for use.

*Capillary press smoking machine*

The automatic machine operates by connecting each of 10 cigarettes, secured in holders around a rotating disc, in turn to a source of vacuum produced by a piston-type arrangement. Standard smoking parameters were used: puff volume 25 ml., puff duration 2 seconds, puff interval 1 minute. The puff of smoke is rapidly forced by the return stroke of the piston through a capillary needle and the resulting condensate is delivered to the dorsal skin of the animal situated about 2 cm., from the tip of the needle. Each animal receives 50 mg., of condensate from 30 puffs to an area of the interscapular region of the dorsal skin which is determined by means of a suitably placed aperture in a small box used as an animal holder.

*4-hour condensate*

Cigarettes were smoked in the automatic smoking machine described by Day (1967) to the same smoking parameters, the condensate being collected in a single cold-trap, diluted with solvent, (acetone/water, 9 : 1 v/v) without evaporation and applied within 4 hours of smoking.

*Mice and details of treatment*

Female, albino mice of a specific pathogen-free strain were obtained from the Pharmaceuticals Division, Imperial Chemical Industries Ltd., at 4–6 weeks of age, allowed to acclimatize for 4 weeks, and randomly allocated to three treatment groups with 100 mice in each group. Hair was removed from the treated area by means of electric clippers immediately before the first treatment and subsequently at weekly intervals.

Group I received 50 mg., condensate from the Capillary Press.

Group II received 50 mg., of 4-hour condensate in 0·3 ml., of solvent applied to the whole length of the dorsal skin.

Group III received 50 mg., of 4-hour condensate in three separate interscapular applications of 0·1 ml., with a 10-second interval between applications, to allow partial evaporation of solvent.

Applications of condensate were made three times per week on Monday, Wednesday and Friday and continued for 56 weeks, when the experiment was terminated and all mice were killed.

The skin of the painted area was examined at weekly intervals throughout the experiment, immediately after shaving, for the presence of skin tumours. All animals in the experiment were examined on the same day by two experienced observers and records of tumour size and site were recorded on individual record cards. For the purposes of this experiment a tumour was defined as a growth arising from the skin surface with a minimum diameter of 2 mm. which persisted for at least 2 weeks.

### RESULTS

There was some variation in mortality between the 3 groups, with a higher rate in Group I, probably associated with the use of restraining boxes, and so the age standardization analysis of Lee (1969, personal communication) has been used to compensate for this variation.

The age standardized numbers of tumour-bearing animals after 56 weeks treatment are given in Table I.

TABLE I.—*Tumour Induction in Mouse Skin After Repeated Application of Cigarette Smoke Condensate of Various Types*

| Group | Condensate | Number of mice | Tumour bearing animals |
|---|---|---|---|
| I | Capillary Press to interscapular area | 100 | 37·3 |
| III | 4-hour to interscapular area | 100 | 42·7 |
| II | 4-hour to whole back | 100 | 18·0 |

Results show that there is no significant difference in mouse skin tumorigenicity between condensates applied within 2 seconds or 4 hours of smoking when equal amounts are applied to a similar area of skin. The Capillary Press probably represents the ultimate in the production of fresh condensate and ought to include some of the volatile and semi-volatile components of tobacco smoke, but in this experiment they appear not to increase the specific activity of the condensate to mouse skin.

Another conclusion reached is that the application of either Capillary Press or 4-hour condensate to the interscapular area is more tumorigenic than the same amount of 4-hour old condensate applied to the whole length of the back. Certainly one explanation of this might be the greater weight of condensate applied per unit area of mouse skin at each application, as shown in Table II.

TABLE II

| Skin site | Number of mice measured | Average area of painted skin | Weight of condensate | Wt./sq. cm. |
|---|---|---|---|---|
| Interscapular | 25 | 7·5 sq. cm. | 50 mg. | 6·7 mg./sq. cm. |
| Whole back | 25 | 21·8 sq. cm. | 50 mg. | 2·3 mg./sq. cm. |

From Tables I and II it can be seen that in this experiment, the skin of the interscapular area compared with the whole back, receives per unit area 2·9 times the amount of condensate, and develops 2·4 times the number of tumour bearing animals. These figures appear to support the relevance of area-density of condensate application in relation to tumorigenicity as a probable factor in quantitative estimates of carcinogenicity requiring further investigation.

### SUMMARY

The specific mouse skin carcinogenicity of cigarette smoke condensate applied within 2–3 seconds of each puff was compared with that of the usual type of condensate applied 4 hours after its production. There was no significant difference in tumorigenicity between these two types of condensate when equal amounts were applied to similar areas of skin. The relationship of area-density of condensate application in such experiments is discussed.

### REFERENCES

DAY, T. D.—(1967) *Br. J. Cancer*, **21**, 56.
SEEHOFER, F., AND HANSZEN, D.—(1965) *Beitr. Tabakforsch.*, **3**, 291.